# Enthalpic Interactions and Solution Behaviors of Solvent-Free Polymer Brushes

**DOI:** 10.3390/polym14235237

**Published:** 2022-12-01

**Authors:** Yi-Ju Chen, Hsiu-Yu Yu

**Affiliations:** Department of Chemical Engineering, National Taiwan University, Taipei 10617, Taiwan

**Keywords:** nanoparticle-organic hybrid materials, molecular dynamics simulations, polymer brush, enthalpic interaction, absorption

## Abstract

We performed molecular dynamics simulations to characterize the role of enthalpic interaction in impacting the static and dynamic properties of solvent-free polymer brushes. The intrinsic enthalpic interaction in the simulation was introduced using different attraction strengths between distinct species. Two model systems were considered: one consisting of binary brushes of two different polymer types and the other containing a mixture of homopolymer brushes and free molecules. In the first system, we observed that, when two originally incompatible polymers were grafted to opposing surfaces, the miscibility between them was significantly enhanced. A less favorable intrinsic enthalpic interaction in the brushes resulted in a more stretched chain configuration, a lower degree of inter-brush penetration, and faster segmental relaxation. In the second system, we characterized the solvent capacity of the homopolymer brushes from variations in the energy components of the system as a function of the number of free molecules. We determined that molecular absorption was driven by the release of the entropic frustration for the grafted chains in conjunction with the chemical affinity between the solutes and polymers. The solute distribution function within the inter-wall space showed that solute–polymer mixing in the middle of the gap occurred preferentially when the enthalpic interaction was more favorable. When this was not the case, absorption was predominantly localized near the grafting surface. From the mean square displacement of the solute, we found that the brush profiles restrained the molecular diffusion perpendicular to the grafting wall; the weaker the attraction from the brush, the higher the solute mobility.

## 1. Introduction

Nanoparticle–organic hybrid materials (NOHMs) are solvent-free nanocomposites consisting of inorganic core particles and tethered polymer chains on the core surface [1,2,3,4,5]. In the absence of solvent, the polymers are subjected to “space-filling” constraints and are treated as incompressible [6,7]. Compared to pure polymers, NOHMs show enhanced thermal, mechanical, and electrochemical stability. The grafted polymers also provide sufficient fluidity despite the absence of solvent. The characteristics of NOHMs enable them to be used in several applications, such as electrolytes in batteries [8,9,10,11,12], lubricants [13,14], or the capturing of carbon dioxide [15,16,17,18,19,20]. The advantage of flexible tunability also enables NOHMs to be easily mixed with other materials. In these applications, NOHMs may consist of a blend of grafted nanoparticles with different corona chemistries [21] or a mixture of polymer-tethered nanoparticles and external molecules [15,16,17]. In this study, the effects of blending polymer-tethered nanoparticles with different chain interactions and inserting free molecules into solvent-free NOHMs are investigated through molecular dynamics (MD) simulations.

At the polymer–polymer interface, the configuration and relaxation dynamics of the grafted polymer chains play a critical role in the applications mentioned above [21,22,23]. To focus on the influences of these properties, we considered an interfacial system of two interacting surfaces with grafted polymer chains (polymer brush system) instead of a bulk system consisting of abundant polymer-tethered nanoparticles. The equilibrium properties of polymer brushes have been a popular theme in simulation studies using MD [24,25,26,27,28,29], Monte Carlo [30,31,32,33], dissipated particle dynamics [34], and Brownian dynamics [35] methods. Among these simulation methods, MD is suitable for studying the monomer dynamics in conjunction with the brush profiles for cases where the hydrodynamic details are negligible. Moreover, MD results have been shown to be consistent with density-functional theory (DFT) predictions in systems of polymer-grafted nanoparticles [36,37] and polymer brushes [38,39]. Compared to all-atom MD simulation, coarse-grained MD simulation is an attractive tool for modeling macromolecular systems because of its increased computational efficiency in accessing the properties of larger length scales and longer time scales. In previous studies, we employed the mean-field DFT [7] and coarse-grained MD [28] to investigate the equilibrium configuration of solvent-free polymer brushes. The effects of mixing brushes of the same chemistry under different grafting densities and chain lengths were elucidated. As the incompressibility in the system has to be maintained, the grafted polymers collaborate to fill the interstitial space uniformly and the corresponding chain configurations are limited. The cooperative space-filling behaviors of the grafted chains naturally lead to “entropic attraction” between them that impacts the chain stretching, brush interpenetration, and segmental relaxation. We demonstrated that, when the polymer chain length was more significant, or the surface grafting density was higher, larger equilibrium inter-wall separation led to more stretched chains, less mixing between brushes, and slower chain relaxation dynamics. In previous discussions of polymers near a surface [40,41,42,43], the entropic attraction was observed to lead to the surface enrichment of chain ends that can reduce the overall free energy. In solvent-free tethered polymers, the entropically driven attraction between the brushes derives from the space-filling requirement and plays a similar role in reducing the free energy. In this study, we investigate the role of enthalpic interactions in mixing two kinds of polymer brushes by explicitly considering different inter-monomer attraction strengths. The coupled entropic space-filling constraint and chemical affinity between distinct monomers may further impact the miscibility of the two polymers.

The solution behaviors of solvent-free brushes reflect the capacity of NOHMs in gas capture applications. NOHMs can absorb CO_2_ by the following mechanisms: the direct enthalpic affinity between functional groups (such as amines or ethylene oxide segments) and CO_2_, or the indirect entropic effect due to free energy reduction when absorbing the molecule into the initially frustrated canopies of polymer chains. Experimental studies have been undertaken to investigate these CO_2_ capture mechanisms by NOHMs [15,16,17,44]. In NOHM-based mixed matrix membranes, gas transport is facilitated by the fluidity of polymers compared to the pure membrane matrix and the selectivity of CO_2_ over other species remains promising [18,19,20]. These observations support the significant role of the brush configuration and relaxation in gas separation using NOHMs. For the absorption of solvent vapor in polymer brushes, the combined grand canonical Monte Carlo–molecular dynamics (GCMC-MD) procedure has previously been implemented to obtain the absorption isotherm over a wide range of relative vapor pressures to effectively characterize the chemical potential of the solvent [45,46,47,48]. As the chemical potential is connected with the free energy change of the system, an alternative approach to access the solution behavior of the brushes would be to analyze the structural characteristics of the interacting brushes to gain the energy information [7,28]. Therefore, we thoroughly examine the distribution functions of the brushes to characterize the system energy. By directly adding a preset number of free particles to the solvent-free system, we quantify the effect of additional molecules on the brush distributions. These free particles may be considered model gas molecules, such as CO_2_ molecules. Therefore, the analyzed energy components of the system for different amounts of added molecules provide useful information on the chemical potential for gas absorption.

The paper is organized as follows: In Section 2, we introduce the models of our brush systems and the simulation methods used. The formulas we apply to analyze the system properties are also explained. Our results are presented in Section 3, divided into two parts. Section 3.1 demonstrates how the configuration and dynamics of solvent-free binary polymer brushes are altered by the inter-species interaction. The miscibility of mismatched polymers is characterized by progressively decreasing the monomer–monomer attraction of one of the polymer types. In Section 3.2, we illustrate the effect of adding free particles (as gas solutes) into the brush systems. The chemical potential of the free particles with varying interaction strength is determined in the dilute limit. Finally, the findings are summarized in Section 4.

## 2. Models and Simulation Methods

**Figure 1:**
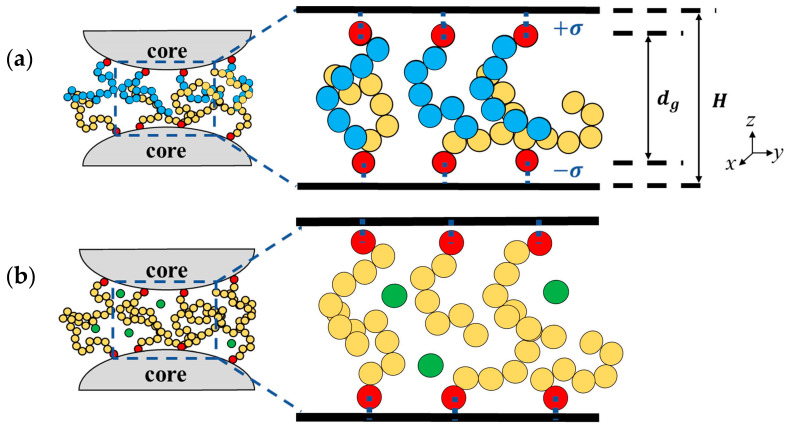
(**a**) Schematic diagram of the binary brush system (right) representing the interstitial space between two neighboring polymer-grafted nanoparticles (left). Red beads are the grafting monomers, and yellow and blue beads are the remaining monomer beads of two types. *H* is the gap thickness and $d_{g}$ is the distance between the top and bottom grafted beads. (**b**) Same as (**a**), but for the same polymer brushes with added free molecules (green beads).

Neglecting the nanoparticle curvature, we consider the grafting of polymer brushes on two opposing flat walls representing the surfaces of two nearby polymer-grafted particles [28]. The two model systems considered in this investigation are shown in the schematic diagrams in Figure 1. In Figure 1a, the interacting brushes composed of different polymers are in a pure, solventless state. In Figure 1b, free molecules are present in the interstices of homopolymer brushes. The interstitial space between the two flat walls is in the *z* direction and periodic boundary conditions are used in the *x* and *y* directions. The polymer chains, each consisting of *N* monomers, are grafted in a uniform square lattice with a grafting density of $\sigma_{g}$. The wall is modeled as a particle-less smooth wall [49] with an area of L×L; the number of chains $N_{c}$ is determined by $N_{c}=\sigma_{g}\times L^{2}$. The bottom wall is located at z=−σ and the grafting monomers are fixed at z=0. The grafting monomers on the top wall are fixed at $z=d_{g}$ and the top wall is located at $z=d_{g}+\sigma$. The gap thickness *H* is the distance between the top and bottom walls ($H=d_{g}+2\sigma$). As the polymers are grafted to inorganic core surfaces, the unfavorable mutual interaction between them is modeled by the repulsive Weeks–Chandler–Andersen (WCA) potential [50],

(1) $$U_{wall}\left(z_{w}\right)=\left\{\begin{array}{cc} 4\varepsilon_{w}\left[{\left(\frac{\sigma_{w}}{z_{w}}\right)}^{12}-{\left(\frac{\sigma_{w}}{z_{w}}\right)}^{6}+\frac{1}{4}\right], & z_{w}\leq z_{w,cut}\\ 0, & z_{w}>z_{w,cut} \end{array}\right.,$$

where $z_{w}$ is the distance between the polymer bead and the wall, $z_{w,cut}=2^{\frac{1}{6}}\sigma_{w}$ is the interaction cut-off distance, and $\sigma_{w}$ and $\varepsilon_{w}$ are the interaction range and strength, respectively. In this model, the first grafting monomer is fixed on the wall and is not affected by this potential.

The attraction between the beads captures the liquid-like characteristics. We consider the pairwise interaction of non-bonding monomer beads and free-particle beads using the Lennard-Jones (LJ) potential of the following form,

(2) $$U_{LJ}\left(r\right)=\left\{\begin{array}{cc} 4\varepsilon_{ij}\left[{\left(\frac{\sigma_{ij}}{r}\right)}^{12}-{\left(\frac{\sigma_{ij}}{r}\right)}^{6}-{\left(\frac{\sigma_{ij}}{r_{cut,ij}}\right)}^{12}+{\left(\frac{\sigma_{ij}}{r_{cut,ij}}\right)}^{6}\right], & r\leq r_{cut,ij}\\ 0, & r>r_{cut,ij} \end{array}\right.,$$

where *r* is the distance between two beads, $r_{cut,ij}=2.5\sigma_{ij}$ is the cut-off distance, and $\sigma_{ij}$ and $\varepsilon_{ij}$ correspond to the bead diameter and the attraction strength, respectively. For the same monomers, i=j. For the adjacent bonding monomer beads, we apply the harmonic bond potential,

(3) $$U_{H}\left(r\right)=k{\left(r-l_{0}\right)}^{2},$$

where $l_{0}=\sigma$ is the equilibrium length of the spring and the spring constant $k=10,000\frac{\varepsilon }{\sigma^{2}}$. The high value is consistent with previous MD studies for grafted chains in the solvent-free condition [28,37,51] and is sufficiently stiff to maintain the bond length at the equilibrium value. We denote the lower polymer brush as type one and the upper as type two. We choose $\varepsilon_{w}=\varepsilon_{11}=\varepsilon =1$ as the unit of energy, while allowing $\varepsilon_{22}$ for polymers two and $\varepsilon_{ff}$ for free particles to vary. The Berthelot mixing rule enables derivation of the LJ potential between the different species: $\varepsilon_{ij}=\sqrt{\varepsilon_{ii}\varepsilon_{jj}}$. For simplicity, we assume that all the beads have identical characteristic sizes, $\sigma_{w}=\sigma_{ij}=\sigma =1$, irrespective of *i* or *j*, and the mass of a bead (*m*) is chosen as 1. On this basis, other length scales, including the gap thickness (*H*) and the polymer radius of gyration ($R_{g}$), have the unit of σ, and the polymer grafting density ($\sigma_{g}$) has the unit of $\sigma^{-2}$. The temperature and pressure are scaled by the LJ parameters as $T^{*}=\frac{k_{B}T}{\varepsilon }$ and $p^{*}=\frac{p\sigma^{3}}{\varepsilon }$, where $k_{B}$ is the Boltzmann constant. It should be noted that employing the Berthelot mixing rule suggests that $\varepsilon_{jj}<\varepsilon_{1j}<1$ would always be true if $\varepsilon_{jj}<1$. Therefore, mixing two species would be intrinsically less favorable for one species but more favorable for the other.

Our MD simulations were performed using the large-scale atomic/molecular massively parallel simulator (LAMMPS) software package [52]. We chose the NVT ensemble with the Nose–Hoover thermostat that maintains the isothermal condition at $T^{*}=1$. The real units of the physical parameters can be determined based on the repeat unit of the chain. Considering a poly(ethylene) glycol chain, for instance, one ethoxy monomer unit $\left(-{\mathrm{CH}}_{2}{\mathrm{OCH}}_{2}-\right)$ leads to $\varepsilon /k_{B}=377$ K, σ=4 Å, and m=44 g mol^−1^ [28,53]. We integrate the equations of motion using the velocity Verlet algorithm at a time step size of δt=0.005τ, with $\tau =\sigma \sqrt{\frac{m}{\varepsilon }}$ being the unit of time. Initially, the two flat walls are sufficiently distant that the grafted polymer brushes do not interact. Then, we slowly move the top wall toward the bottom to reach a specific gap thickness allowing the polymers to interpenetrate. At a fixed gap thickness, we first perform $10^{6}$ time steps of the equilibration run followed by $5\times 10^{6}$ time steps of the production run and collect the thermodynamic data every 100 time steps. The collected data are averaged to check if the pressure reaches the atmospheric pressure (corresponding to $(p^{*}\sim 0.001$). If not, we adjust the gap thickness, recollect the thermodynamic data, and repeat the pressure calculation procedure until the target pressure is met. This equilibration process applies to both model systems in this investigation. When free particles are present, the prescribed number of free particles are randomly inserted into the originally equilibrated brushes. The top wall is then allowed to move for the system to again reach the atmospheric condition.

As mentioned in the Introduction, previously, we demonstrated that the entropic effects of varying the chain length and the grafting density were similar in solvent-free brushes [7,28]. Throughout this investigation, when comparing the intrinsic space-filling capability of polymers, we change the chain length at a fixed grafting density. For interacting monodisperse brush systems at the same reduced temperature and pressure, it has been found that the chain configuration is close to the reference melt state if N=32 and $\sigma_{g}=0.125$ [28]. Therefore, we choose this parameter space in most brush systems investigated and vary the LJ interaction strength for one of the brushes. The number of polymers per wall is fixed at $N_{c}=1600$. In Table 1, the classification for the brush type is listed. All the systems have the same grafting density and brushes A to J have the same chain length. Given the listed brush type, system AA denotes the monodisperse system where both walls are grafted with the same brush of type A, while system AB represents the binary system where the bottom wall is of brush type A and the top wall is B. In the presence of free particles, we choose systems AA and MM as the pure reference systems and change $\varepsilon_{ff}$ of the free particles. We denote these systems AA_$\varepsilon_{ff}$ or MM_$\varepsilon_{ff}$.

After the target equilibrium pressure is obtained, the structural and dynamical properties of the system are analyzed. We calculate the bead density distribution ($\rho_{b}\left(r\right)$) and local volume fraction of the beads (ϕ(r)) to characterize the equilibrium morphology of the system using the equilibrated $5\times 10^{6}$ time steps with the data being collected every 1000 time steps. The volume fraction is defined by

(4) $$\phi \left(r\right)=\int_{}^{}\rho_{b}\left(r-r^{\prime }\right)\Pi \left(\left|r^{\prime }\right|-\frac{\sigma }{2}\right)dr^{\prime },$$

where $\Pi \left(\left|r\right|-\frac{\sigma }{2}\right)$ is the ball function that limits the volume contribution within a distance of $\frac{\sigma }{2}$ from a given position. The symmetry of the brush system in both *x* and *y* directions makes $\phi \left(r\right)=\phi \left(z\right)$. By plotting ϕ against *z*, we determine the degree of interpenetration between two brushes *P* by calculating the ratio of the the intersection of the volume fractions for the top and bottom brushes to the total volume fraction of the two brushes, $P=\left[\int \left(\phi_{u}\cap \phi_{l}\right)dz\right]/\left[\int \left(\phi_{u}+\phi_{l}\right)dz\right]$, with $\phi_{u}$ and $\phi_{l}$ being the volume fraction distributions of the upper and lower brushes, respectively.

The bead distribution functions directly determine the energy state of the brushes. Provided the bead density relative to the grafting surface, we characterize the configurational entropy of the tethered polymer relative to a homogeneous polymer melt at the same average bead density as [28],

(5) $$S^{*}=\frac{S}{nk_{B}}=-\frac{1}{H}\int_{0}^{H}g(z)\mathrm{lng}\left(z\right)\;dz.$$

$S^{*}$ denotes the scaled excess entropy per bead, *n* is the total number of beads in the brush, and g(z) is the bead distribution function calculated as the ratio of $\rho_{b}\left(z\right)$ to the average bead number density in the gap.

We obtain the excess internal energy of the monomer bead directly by subtracting $3T^{*}/2$ from the total energy output of LAMMPS, which essentially is the potential energy contribution. With the values of the excess configurational entropy and excess internal energy, the excess free energy per bead is calculated by the fundamental relation,

(6) $$F^{*}=U^{*}-T^{*}S^{*}.$$

For systems with free particles added, the excess entropy contribution of the free particle within the brush is again calculated by Equation (5) with the particle distribution function being determined relative to the bottom wall. The associated excess internal energy and the excess free energy are determined in the same manner as for the polymers. Additionally, we characterize the free energy profile (potential of mean force) of a chosen free particle along the gap thickness in the *z*-direction. The Helmholtz free energy difference of the state with the free particle being located at $z_{i}$ from a reference state can be calculated as [54,55,56]

(7) $$\Delta F\left(z_{i}\right)=-\int_{z_{0}}^{z_{i}}\langle f_{z}\left({\bar{z}}_{i}\right)\rangle \;d{\bar{z}}_{i},$$

where ${\bar{z}}_{i}$ is the average position of the particle and $f_{z}$ is the *z*-component of the total force exerted on that particle. When determining this force, we apply a spring force against the motion of the chosen free particle. In equilibrium, we back-calculate the mean force exerted on the particle from the monitored displacement of the particle. Owing to the symmetry of our system, we take $z_{0}$ as the center of the gap ($z_{0}=\frac{H}{2}$), and we choose to only integrate from the center to the bottom wall.

The dynamical properties of interest include the autocorrelation function and mean square displacement of the chain end. These properties allow for a straightforward characterization of the segmental relaxation of the tethered polymer. As the polymers are grafted and confined within the gap, instead of monitoring the actual position of the chain end, it would be more appropriate to analyze the instantaneous position of the chain end relative to its equilibrium position, namely, the end-to-end (or chain-end) fluctuation. Consequently, we calculate the normalized end-to-end fluctuation autocorrelation function as

(8) $$C_{A,\alpha }\left(t\right)=\langle A_{\alpha }\left(0\right)A_{\alpha }\left(t\right)\rangle /\langle A_{\alpha }^{2}\rangle ,$$

where $A_{\alpha }\left(t\right)$ is the end-to-end fluctuation (end-to-end distance relative to the equilibrium value) with α=x, *y*, or *z*. The fluctuation autocorrelation function is a way of observing how the instantaneous position is correlated with the initial equilibrium condition, and we apply $\langle A_{\alpha }\left(t_{1}\right)A_{\alpha }\left(t_{2}\right)\rangle =\langle A_{\alpha }\left(0\right)A_{\alpha }\left(t\right)\rangle$ for $t_{2}-t_{1}=t$ at thermal equilibrium. Here, $\langle A_{\alpha }\rangle$ is the ensemble average of $A_{\alpha }$ over time and samples. We apply time shifting when calculating the fluctuation autocorrelation functions to achieve satisfactory statistics. Given the definition of the end-to-end fluctuation relative to the equilibrium value, namely, the displacement of the end-to-end fluctuation at thermal equilibrium,

(9) $$\begin{array}{cc} MSF_{A,\alpha }\left(t\right) & =\langle {{(A}_{\alpha }\left(t\right)-A_{\alpha }\left(0\right))}^{2}\rangle =\langle {A_{\alpha }\left(t\right)}^{2}\rangle -2\langle A_{\alpha }\left(t\right)A_{\alpha }\left(0\right)\rangle +\langle {A_{\alpha }\left(0\right)}^{2}\rangle \\ \; & =2\langle {A_{\alpha }\left(0\right)}^{2}\rangle -2\langle A_{\alpha }\left(t\right)A_{\alpha }\left(0\right)\rangle , \end{array}$$

We also relate the mean square fluctuation and the autocorrelation function by

(10) $$MSF_{A,\alpha }\left(t\right)=2\langle {A_{\alpha }}^{2}\rangle \left(1-C_{A,\alpha }\left(t\right)\right),$$

where $\langle {A_{\alpha }}^{2}\rangle =\langle {A_{\alpha }\left(0\right)}^{2}\rangle$ is the variance of the distribution of the end-to-end fluctuation in equilibrium. Therefore, $C_{A,\alpha }\left(t\right)$ is affected by both $MSF_{A,\alpha }\left(t\right)$ and $\langle {A_{\alpha }}^{2}\rangle$ following [28]

(11) $$1-C_{A,\alpha }\left(t\right)=\frac{MSF_{A,\alpha }\left(t\right)}{2\langle {A_{\alpha }}^{2}\rangle }.$$

For the free particles, the dynamical behavior is presented in terms of the mean square displacement analyzed in the standard manner. The ensemble average for dynamical analysis is based on the production run performed at the equilibrated gap thickness for $2.5\times 10^{7}$ time steps with the data collected every 1000 time steps.

## 3. Results And Discussion

### 3.1. Properties of Solvent-Free Binary Polymer Brushes

To gain insight into the mixing behaviors of solvent-free binary brushes, we first characterize the enthalpic interaction between two kinds of grafted polymer species in terms of the polymer structural properties, system energies, and segmental dynamics. For structural properties, we calculate the local volume fraction distribution of the polymer beads (ϕ), the degree of interpenetration of the two opposing brushes (*P*), and the ratio of the average *z*-component of the actual gyration radius to the reference finite-length freely-jointly chain (FJC) result ($R_{g}^{0}$) [57], $R_{gz}^{*}=R_{gz}/R_{g}^{0}$. For the system energies relative to the ideal state, we calculate the scaled internal energy per bead ($U^{*}$), the scaled configurational entropy per bead ($S^{*}$), and the scaled free energy per bead ($F^{*}$) of each polymer species. These equilibrium properties are generally compared for different values of the scaled gap thickness, $H^{*}=H/R_{g}^{0}$. For segmental dynamics, we characterize the probability distribution functions of the end-to-end fluctuation ($P(A_{\alpha })$), the mean square displacements of the end-to-end fluctuation ($MSF_{A,\alpha }\left(t\right)$), and the normalized end-to-end fluctuation autocorrelation functions ($C_{A,\alpha }\left(t\right)$) in both *x* (parallel to the wall) and *z* (perpendicular to the wall) directions.

**Figure 2:**
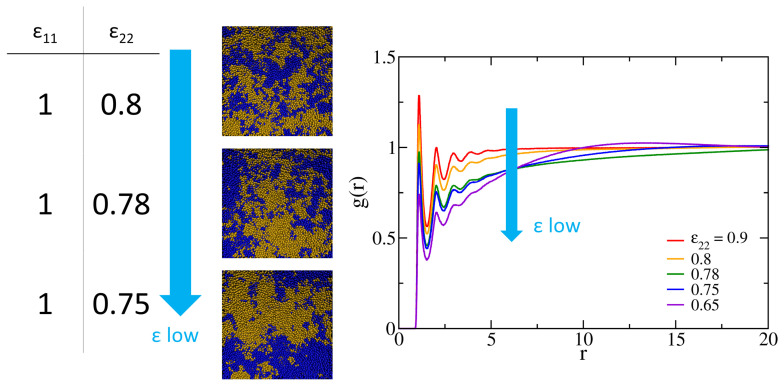
Left: simulation snapshots of binary blend systems at different values of the LJ potential well for the second polymer type. Chains of two colors denote two different polymer types. Right: the simulated radial distribution function between monomer beads for various $\varepsilon_{22}$ based on the one-fluid approximation [59].

Before separately grafting polymers one and two to the surfaces, it is of interest to first investigate the miscibility of the polymer blend of the two kinds. The Flory–Huggins interaction parameter measures the free energy cost of placing two disliked monomers next to each other. The phase separation occurs for a binary blend with $\chi >\chi_{c}$ with $\chi_{c}$ being the critical value. The theoretical $\chi_{c}$ depends on the chain length and $\chi_{c}N=2$ for the equal-length, symmetric polymer mixture [58]. In order to gain insight into how the interaction parameter depends on the LJ potentials and further compare the miscibility of different polymers at the melt and grafted states, we conduct a series of NVT simulations at the atmospheric condition for the blend of 1600 chains of each polymer species (3200 chains in total) with 32 monomers per chain. By decreasing $\varepsilon_{22}$ of polymer two from 1 while maintaining $\varepsilon_{11}=1$, we observe that phase separation occurs roughly when $\varepsilon_{22}<0.8$, as evidenced by the morphological changes in Figure 2. Clumps of blue and yellow regions apparently grow as $\varepsilon_{22}$ decreases from 0.8 to 0.75. For binary polymer mixtures, the Flory–Huggins interaction parameter χ can be related to the corresponding LJ potential wells ($\varepsilon_{11}$, $\varepsilon_{22}$, and $\varepsilon_{12}$) through the mean-field approximation [59],

(12) $$\chi =\frac{\rho }{2k_{B}T}\int_{0}^{r_{c}}\left[2U_{12}\left(r\right)-U_{11}\left(r\right)-U_{22}\left(r\right)\right]\;g_{12}\left(r\right)dr,$$

where $U_{ij}$ is the LJ potential between species *i* and *j* and $g_{12}\left(r\right)$ is the radial distribution function of the binary mixture, taking into account only the contributions between monomer beads from different chains. Making use of this expression, we estimate $\chi_{c}$ for our system by evaluating the radial distribution function using two methods: (1) analytically approximating $g_{12}\left(r\right)\approx \exp\left(-U_{12}/k_{B}T\right)$, and (2) computing the one-fluid g(r) from the simulations, irrespective of the polymer type. As shown in Figure 2, as $\varepsilon_{22}$ decreases, the first peak in g(r) decreases accordingly. When the first peak decreases to 1 (roughly at $\varepsilon_{22}=0.78$), correlations at a larger length scale develop, and the dominant peak gradually shifts to larger *r*. This behavior of peak transition suggests phase separation and is consistent with the phase growth shown in the snapshots of Figure 2. The correlations corresponding to the developed peak come from the segregated structures. Therefore, we choose $\varepsilon_{22}=0.78$ as the critical attraction strength. Based on this choice, it is expected that g(r) obtained from the demixing system would provide a lower bound of $\chi_{c}$ as the nearest-neighbors correlations are reduced. At $\varepsilon_{22}=0.78$, the analytical approximation leads to $\chi_{c}^{ana}\approx 0.0819$ and the simulation yields $\chi_{c}^{sim}\approx 0.0335$. The theoretical result of $\chi_{c}=2/N=0.0625$ is indeed bounded by the two values, suggesting that our mean-field estimate of the critical $\varepsilon_{22}^{c}$ for the binary blend of 32-mers is consistent with the Flory–Huggins theory.

**Figure 3:**
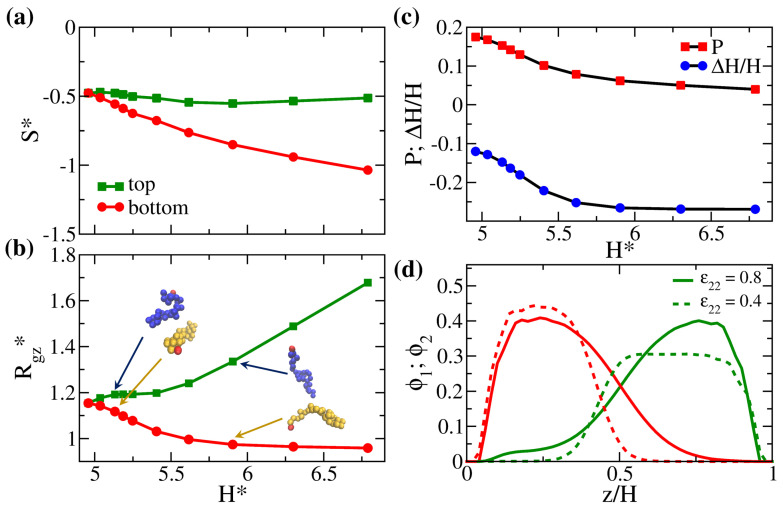
(**a**) Scaled entropy per bead (S*), (**b**) scaled average *z*-component radius of gyration ($R_{gz}^{*}$), and (**c**) degree of the interpenetration of polymers from two walls determined from different measures (*P* and ΔH/H; see text) plotted against the scaled gap thickness ($H^{*}=H/R_{g}^{0}$) for systems AA, AB, AC, AD, AE, AF, AG, AH, AI, and AJ (symbols from left to right). (**d**) Local volume fraction distributions of the system AC ($\varepsilon_{22}=0.8$) and the system AH ($\varepsilon_{22}=0.4$). The green lines are the profiles for the top brush and the red curves correspond to the profiles for the bottom brush. For clarity, the corresponding representative snapshots of a pair of grafted chains are shown in (**b**).

The mixing characteristics are altered when the mismatched polymers are separately grafted to two surfaces. Given the fixed grafting density of $\sigma_{g}=0.125$, we perform simulations on the binary brush systems of AA, AB, AC, AD, AE, AF, AG, AH, AI, and AJ (see Table 1 for the classification), where we fix $\varepsilon_{11}=1$ for the bottom brush A and $\varepsilon_{22}$ for the top brush decreases from 1 to 0.2. The excess internal energy is expected to increase as the contrast of interaction ($\Delta \varepsilon =\varepsilon_{11}-\varepsilon_{22}=1-\varepsilon_{22}$) increases. Instead, we focus on the structural variations in these solvent-free brush systems to elaborate the changes in the configurational space. As Δε increases, the overall monomer–monomer attraction in the system decreases, which leads to an increase in the gap thickness. Therefore, in Figure 3 we present the scaled configurational entropy, the chain stretching, and the degree of interpenetration against $H^{*}$ to bring out the space-filling effect at different strengths of attraction. The increase in the gap thickness is primarily caused by the decrease in the intra-brush attraction for the upper brush. As Δε becomes larger, a greater fraction of the interstitial space in the upper brush is filled with the “phantom solvent” [37]. Therefore, compared with the lower brush with strong monomer–monomer attractions, the upper brush is in a relatively good solvent condition at the given temperature and pressure. The configurations of the two brushes vary differently with the change in $H^{*}$. As seen in Figure 3, although the upper brush two generally becomes more extended, the lower brush one gradually collapses as the inter-brush attraction is weaker than the intra-brush attraction for the lower brush, i.e., $\varepsilon_{12}<\varepsilon_{11}$ given our definition of $\varepsilon_{12}=\sqrt{\varepsilon_{11}\varepsilon_{22}}$. Specifically, in Figure 3b, when $H^{*}$ increases, $R_{gz}^{*}$ for polymer two first reaches a plateau at small Δε followed by a more substantial increase at large Δε. Nevertheless, $R_{gz}^{*}$ for polymer one first decreases more rapidly at small Δε and levels off at large Δε.

The two-stage variations in the chain configurations of the two brushes suggest that the polymers cooperate to fill the space. In Figure 3d, we compare the profiles for the AC and AH systems, corresponding to systems with small and large values of Δε, respectively. It can be seen that, when $\varepsilon_{22}=0.8$ (AC system), the two brush profiles are quite symmetric, with a tail region for brush C extending to the opposite wall, allowing sufficient mixing between the two polymers. In contrast, when $\varepsilon_{22}$ is as small as 0.4 (AH system), brush H displays a highly stretched profile while brush A collapses to limit the inter-brush mixing. The variations of the chain configuration are also illustrated in the representative snapshots for a pair of grafted polymers in Figure 3b. Even for such asymmetric brush profiles, a portion of the brush still penetrates the opposing one. From the variation in the degree of interpenetration in Figure 3c, apparently, as $H^{*}$ increases with Δε, *P* decreases accordingly. In order to more directly compare how the chain configuration impacts the inter-brush mixing, we introduce a second indicator to quantify the degree of interpenetration: $\Delta H=H-R_{ez,1}-R_{ez,2}$, where $R_{ez,i}$ is the average end-to-wall distance of the polymer *i* with i=1 or 2. We observe that the normalized ΔH/H shows the same trend as *P*. Similar to $R_{gz}^{*}$ for polymer one, ΔH/H eventually flattens as $\varepsilon_{22}<0.5$. As Δε is significant, the increase in the gap thickness is indeed attributed to the stretching of polymer two while the degree of interpenetration almost remains constant. Finally, in Figure 3a, as $H^{*}$ increases, the scaled configurational entropy of the lower brush decreases monotonically because the brush configuration becomes more and more limited. Conversely, the competition between the effect of inter-brush mixing and chain stretching in the presence of the phantom solvent yields subtle variation in the entropy of polymer two.

The overall observations in Figure 3 illustrate the unique mixing characteristics of solvent-free binary brushes. If we choose Δε=0.55 (between systems AG and AH) as the critical interaction contrast for mixing, above which the brushes show a nearly constant degree of interpenetration, then the corresponding $\varepsilon_{22}=0.45$ is significantly smaller than $\varepsilon_{22}=0.78$ for the blended polymer melt. In other words, the space-filling constraint in the systems facilitates the mixing of two disliked brushes and delays the phase separation.

**Figure 4:**
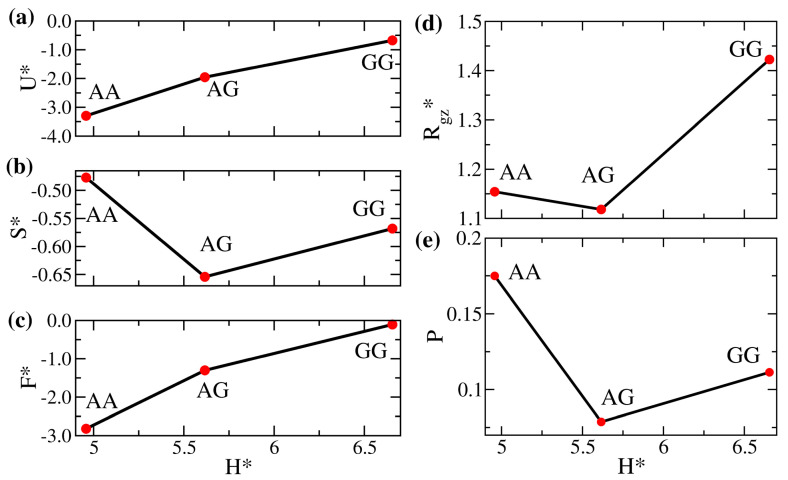
(**a**) Scaled internal energy per bead ($U^{*}$), (**b**) scaled entropy per bead ($S^{*}$), (**c**) scaled free energy per bead ($F^{*}$), (**d**) ratio of the *z*-component actual gyration radius to the reference gyration radius ($R_{gz}^{*}$), and (**e**) degree of the interpenetration of polymers (*P*) versus the dimensionless gap thickness ($H^{*}$) of AA, AG, and GG systems.

**Figure 5:**
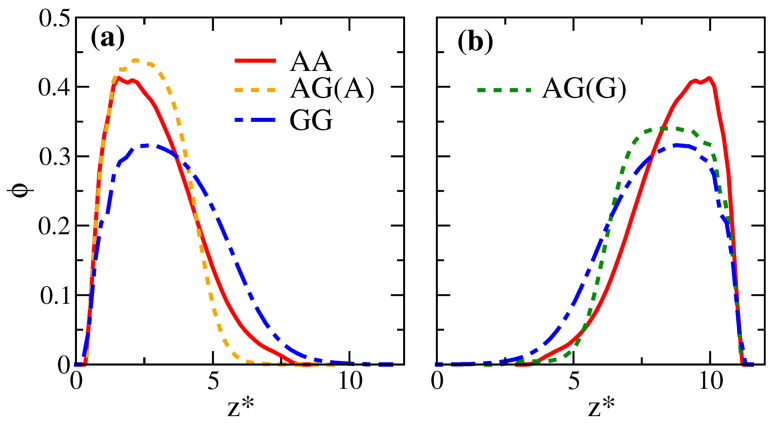
Local volume fraction distributions of the polymer beads (ϕ) plotted along the scaled $z^{*}=z/R_{g}^{0}$ position for systems AA, AG, and GG. The distributions of the bottom brush are shown in (**a**); those for the upper brush are shown in (**b**). The dashed curves in (**a**,**b**) correspond to the profiles of brush A and brush G in the binary system, respectively. For clarity and easy comparison, the brush profiles in (**b**) are shifted horizontally to make all curves start from the same $z^{*}$.

After characterizing the overall miscibility of the enthalpic solvent-free binary brushes, we examine how the properties of the mixed brushes are compared with their corresponding pure counterparts. To more clearly elaborate the impact of mismatched interactions, in Figure 4, we compare the equilibrium properties for systems AA, AG, and GG as a function of the gap thickness in the unit of $R_{g}^{0}$, $H^{*}=H/R_{g}^{0}$. The AA and GG systems are homogeneous brushes, and system AG is the corresponding binary mixture. The system GG has the weakest overall attraction (smallest $\varepsilon_{ij}$), resulting in the highest internal energy in Figure 4a and the largest inter-wall spacing among the three systems. As the chains uniformly fill the space, this increased gap thickness causes system GG to show a more stretched polymer chain configuration. As already indicated in Figure 3, the chains are as if immersed in a good solvent, leading to the largest $R_{gz}^{*}$ in Figure 4d. This structural characteristic is also displayed in the brush profiles in Figure 5, where the distribution for the GG brush is generally broader. The strong mismatch between the brushes A and G ($\varepsilon_{11}=1$ vs. $\varepsilon_{22}=0.5$) makes their distributions in the mixed state highly distinct compared with their pure states. The unfavorable interaction yields the lowest inter-brush mixing in Figure 4e, the smallest average chain stretching in Figure 4d, and the smallest bead configurational entropy in Figure 4b among the three systems. Although $S^{*}$ varies non-monotonically, the excess free energy per bead remains the highest for the system GG in Figure 4c. The variations in these equilibrium properties become less significant if the interaction contrast (Δε) is reduced to be less than 0.3 (roughly corresponding to the systems with $H^{*}<5.3$ in Figure 3). In the Supplementary Materials, the results for systems AA, BB, and AB (with a smaller Δε=0.1) are compared. As presented in Figures S1 and S2, the associated structural changes are more modest, and the brush profiles are slightly different among the three systems.

**Figure 6:**
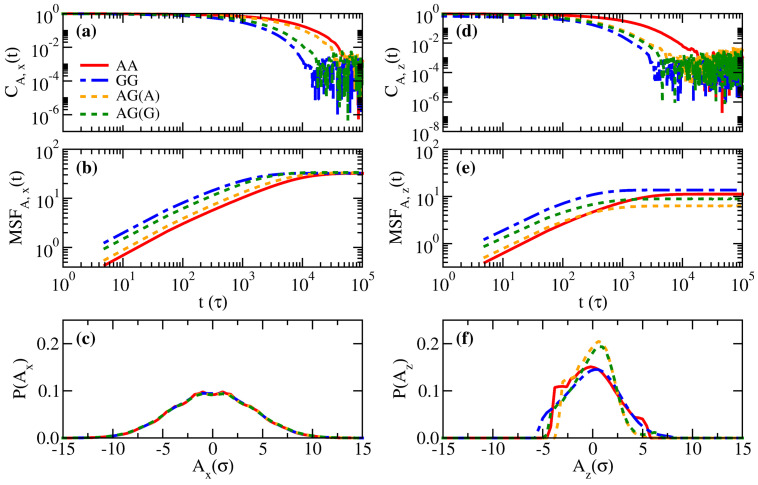
Comparisons of the normalized end-to-end fluctuation autocorrelation functions ($C_{A,\alpha }\left(t\right)$), the mean square displacements of the end-to-end fluctuation (${MSF}_{A,\alpha }\left(t\right)$), and the probability distribution functions of the end-to-end fluctuation ($P(A_{\alpha })$) for systems AA, AG, and GG in the parallel direction, (**a**–**c**), and the perpendicular direction, (**d**–**f**).

Next, we examine the end-to-end relaxation dynamics for systems AA, AG, and GG in Figure 6. The three essential properties mentioned in Equation (11) are summarized, namely, the fluctuation autocorrelation functions ($C_{A,\alpha }\left(t\right)$), the mean square displacements of the fluctuations ($MSF_{A,\alpha }\left(t\right)$), and the probability distribution of the fluctuation ($P(A_{\alpha })$). First, as the strong inter-monomer attraction tends to maintain a nearly constant local monomer volume fraction, it is expected that such a restriction would slow down the relaxation of polymers. Therefore, Figure 6a,d suggest that the stronger the average attraction between monomers, the slower the temporal decay in the fluctuation autocorrelation functions. Although the curves of $C_{A,x}\left(t\right)$ for different systems are well-separated, $C_{A,z}\left(t\right)$ for brush A in the binary system is obviously close to that for brush G, suggesting that the fast segments facilitate the relaxation of the slow segments. This observation is consistent with the previous finding in purely entropic binary brushes where longer (and slower) polymers conform with the relaxation of shorter (and faster) ones [28]. The distributions of the end-to-end fluctuations in the parallel direction in Figure 6c are broad and symmetric, irrespective of the brush type. However, irregular and narrower profiles of $P(A_{z})$ are seen in Figure 6f, which indicates the distortion of the end-to-end fluctuation due to the confinement in the perpendicular direction. The trends in $MSF_{A,\alpha }\left(t\right)$ (Figure 6b,e) are generally dependent on $C_{A,\alpha }\left(t\right)$ and $P(A_{\alpha })$ through the relations in Equations (10) and (11). As the profiles of $P(A_{z})$ for the AG system are narrower than the AA and GG systems, it is not surprising that the mean square displacements of the fluctuations perpendicular to the wall for the AG system are smaller than for the AA and GG systems. All the outlined effects are less significant if we change the opposing brush to brush B, as shown in Figures S3–S5.

### 3.2. Solution Behaviors of Polymer Brushes

Once the enthalpic effect of mixing the two brushes is characterized, we investigate the solution behavior of the brushes by including a fraction of another species. In this section, we consider a binary mixture of free particles (as gas solutes) and polymer brushes to first understand the absorption characteristics of the brushes at equilibrium. For solvent-free brushes, the space-filling constraint limits the available chain configurational space and the corresponding entropy. The additional solutes help to fill the interstitial space, which then releases the entropic frustration of polymers—a thermodynamic effect that may drive the gas absorption.

We vary the number of solute particles in the system to explore how the solute concentration impacts the system properties. Given the number of free particle beads ($N_{f}$), the fraction of free particles ($n_{f}$) is defined as the number of free particle beads over the total number of beads in the system ($N_{f}/\left(N_{f}+2N_{c}N\right)$). In order to independently elaborate the entropic and enthalpic effects on solute uptake, we first change the chain length of polymers while fixing the interaction strength of the free solute particles as $\varepsilon_{ff}=1$. After that, we specify the polymer length and consider the solute–solute interaction ($\varepsilon_{ff}$) as a variable. The energy components focused on include the scaled configurational entropy per bead ($S^{*}$) for polymer brushes, the scaled free energy per bead for polymer brushes ($F^{*}$), and the scaled total free energy per bead for the system ($F_{t}^{*}$). The structural properties reported include the local volume fraction distributions, the chain stretching characterized by the ratio of the *z*-component radius of gyration over the reference FJC value, and the degree of inter-brush penetration. For clarity, the values of these properties are also reported in Tables S1–S8 in the Supplementary Materials.

**Figure 7:**
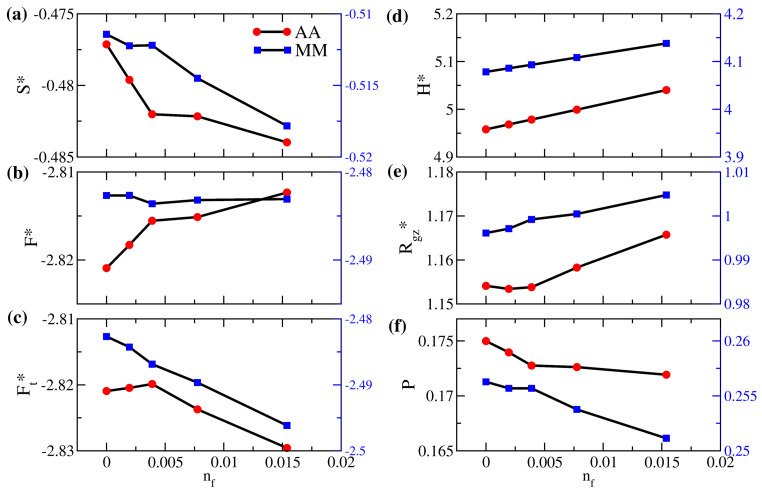
(**a**) Scaled entropy per bead ($S^{*}$), (**b**) scaled free energy per bead for polymer brushes ($F^{*}$), (**c**) scaled total free energy per bead ($F_{t}^{*}$), (**d**) scaled gap thickness ($H^{*}$), (**e**) degree of interpenetration of polymers (*P*), and (**f**) ratio of the *z*-component actual gyration radius to the reference gyration radius ($R_{gz}^{*}$) as a function of the number fraction of free particles ($n_{f}$) for brushes AA (red circles; left axis) and MM (blue squares; right axis). The two vertical axes are fixed at the same range of values.

**Figure 8:**
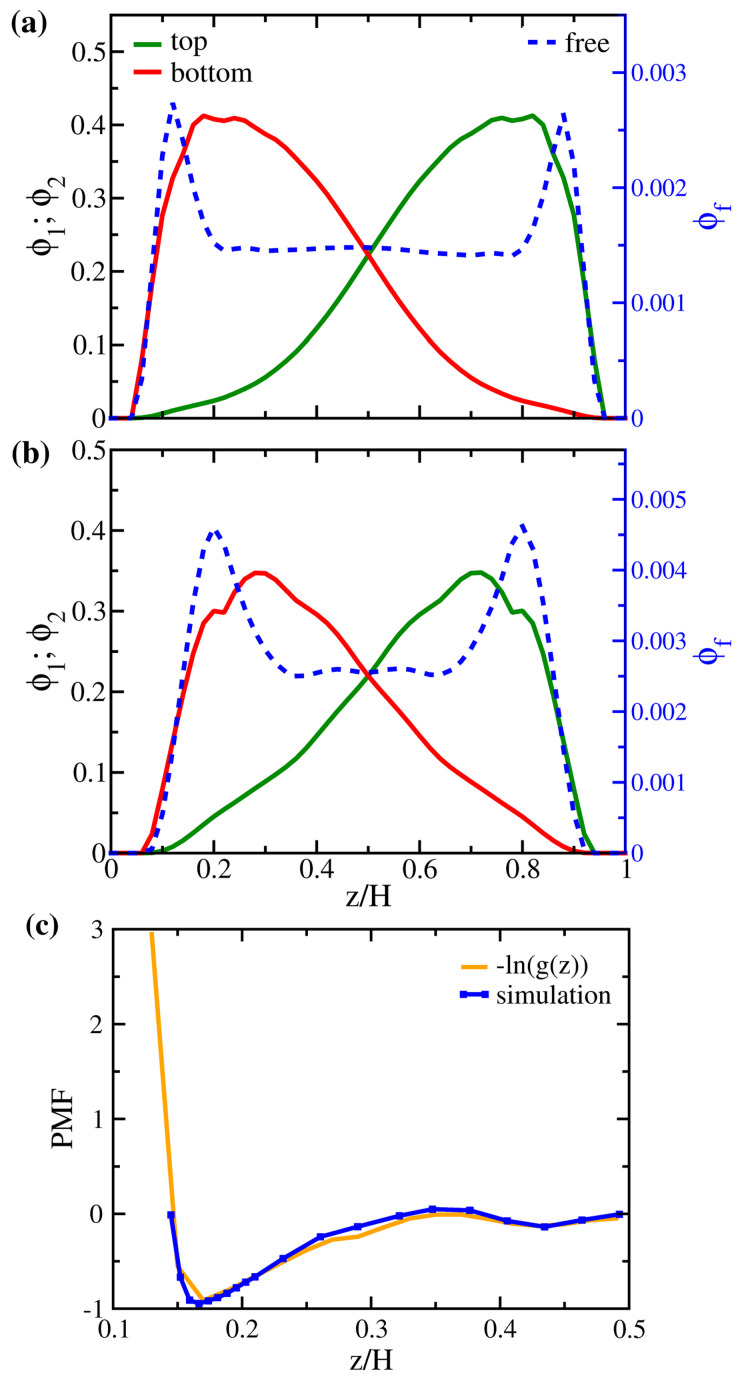
Local volume fraction distributions of polymer beads (solid lines; left axis) and free particles (dashed line; right axis) for (**a**) AA+400 and (**b**) MM+400. (**c**) Comparison of the potential of mean force calculated from g(z) and directly from simulation (see text in Section 2) for system MM+400. The energy at z/H=0.5 is set to zero as a reference.

In Figure 7 and Figure 8, we first investigate the variations in the energy components and the structural properties due to free particles of the same interaction for systems AA and MM. This model system of $\varepsilon_{ff}=1$ would be a pertinent reference to illuminate the entropic effect when free solutes are captured. This condition also applies to cases where the absorbed species have very similar chemistry to the monomer molecules. First of all, Figure 7 indicates that, as the number fraction of free particles increases, polymers in the AA system generally have a more stretched configuration with an increasing $R_{gz}^{*}$ (in (e)) as a result of the increased gap thickness (in (d)). As free particles fill the interstitial space, the interpenetration between brushes in (f) gradually decreases, leading to a reduction in the entropy per bead in (a). Since the variation in the internal energy is only minor, the decrease in the entropy yields an increase in the free energy per bead of the AA brushes in (b). Nevertheless, if we closely examine the range of $n_{f}<0.005$ in the dilute limit, we observe that the variations of the properties for brushes AA are different. Specifically, when the number fraction of added particles is less than 0.00389 (corresponding to 400 free particles added), free particles fill the middle of the gap, immediately reducing the end-to-wall distance of polymers. This initial effect decreases $R_{gz}^{*}$ and makes *P* drop noticeably. When $n_{f}$ is over 0.005, the “free volume” at the polymer–wall interface due to grafting attracts more free particles dispersed in the near-wall region, as shown in Figure 8a, and the polymer configuration adjusts differently from the dilute limit. The overall effects lead to a non-monotonic variation in the total free energy per particle of system AA in Figure 7c. Since the configuration of the solvent-free AA polymers is closer to the reference polymer melt, a more favorable thermodynamic state, a small number of additional free particles disturb this favorable state of the system, leading to an increase in energy. The bell-like local volume fraction profile for brushes AA in Figure 8a is consistent with the ideal brush state. When more free particles are included, mixing brushes and particles of the same attraction is generally favorable and the total free energy per particle is reduced. In our NVT simulations, the minimal variation in the gap thickness at equilibrium makes the PV work negligible. Therefore, we approximate the chemical potential of free particles as $\mu ={\left(\frac{\partial G}{\partial N_{f}}\right)}_{T,P,n}\approx {\left(\frac{\partial F}{\partial N_{f}}\right)}_{T,P,n}$, accounting for the change in the total system free energy due to inserting or deleting one free particle. In the dilute limit with $n_{f}<0.005$, the calculation yields a chemical potential of −2.58 for the free solute in brushes AA. This negative value of the chemical potential indicates a driving force of absorption resulting from both the initial release of the entropic frustration of grafted polymers as $H^{*}$ increases and the “absorption site” at the polymer grafting points. As the number fraction of solutes is only moderate in our study, the segmental dynamics of the polymer chains are almost unaffected by the presence of free particles. The main characteristics presented in Figures S6–S8 remain unchanged from those observed in the solvent-free AA system.

Compared to the nearly-ideal conformational state for the polymers in system AA, in Figure 8b, the shorter polymers in system MM exhibit more variations in the local volume fraction near the grafting surface, suggesting a less favorable thermodynamic state. As a result, it is anticipated that, for shorter and more intrinsically frustrated tethered polymers, such as those in system MM, all the entropic effects on solute uptake would be different. It turns out that an increase in the number fraction of free particles in system MM yields more moderate initial variations in the interpenetration of polymer brushes in Figure 7f and the associated entropy per bead in Figure 7a; $R_{gz}^{*}$ now changes monotonically without a minimum (Figure 7e), and the trends in the free energy components (Figure 7b,c) are distinct from system AA. When free particles are inserted, the increased inter-wall separation ($H^{*}$) makes the originally compressed brushes (with $R_{gz}^{*}<1$) relax more prominently and mix with the free particles. This effect leads to a consistent increase in $R_{gz}^{*}$ and reduces the corresponding impact of the higher gap thickness on the brush interpenetration. Therefore, as more solute particles are added in system MM, the brush free energy per bead varies slightly, whereas the total free energy per bead decreases monotonically. From the free energy data in the dilute condition, we calculate the chemical potential of the free particle in system MM as −3.30. Compared with system AA, this value suggests that the solvent-free brushes with shorter polymers may have a stronger driving force for solute or gas uptake resulting from the release of stronger entropic frustration of polymers.

To investigate the potential energy landscape of the free particles among the brushes, we choose the system MM+400 (400 free particles added to the brushes MM) as the representative and calculate the potential of the mean force (PMF) of a single free particle along the *z*-direction using Equation (7). For a bulk fluid, the PMF is related to the radial distribution function in terms of a Boltzmann factor [60]. In the same spirit, the PMF of the free particle at *z* can also be defined by the corresponding distribution function via $PMF=-k_{B}T\ln\left(g\left(z\right)\right)$. As $k_{B}T=1$ in our simulations, we simply compute −ln(g(z)) and compare the result to the PMF in Figure 8c. The close agreement between the two profiles not only justifies the correctness of our simulation protocols but also implies that the distribution of free particles is directly governed by the potential field felt by them within the gap. The two potential wells around z/H=0.17 and z/H=0.43 in Figure 8c correspond to the near-wall grafting site and the mixing region in the center of the gap, respectively. This again suggests that two entropic factors drive the uptake of solutes—packing in the free volume at the polymer–surface interface and the polymer–solute mixing at the chain ends.

Finally, we adjust the interaction strength of the added particles in the AA system to explore the effect of enthalpic interaction between the absorbed molecules and brushes. Aside from the reference case of $\varepsilon_{ff}=1$, we consider the conditions of $\varepsilon_{ff}=0.5$ and $\varepsilon_{ff}=1.5$, and denote the three systems as AA_1.0, AA_0.5, and AA_1.5, respectively.

**Figure 9:**
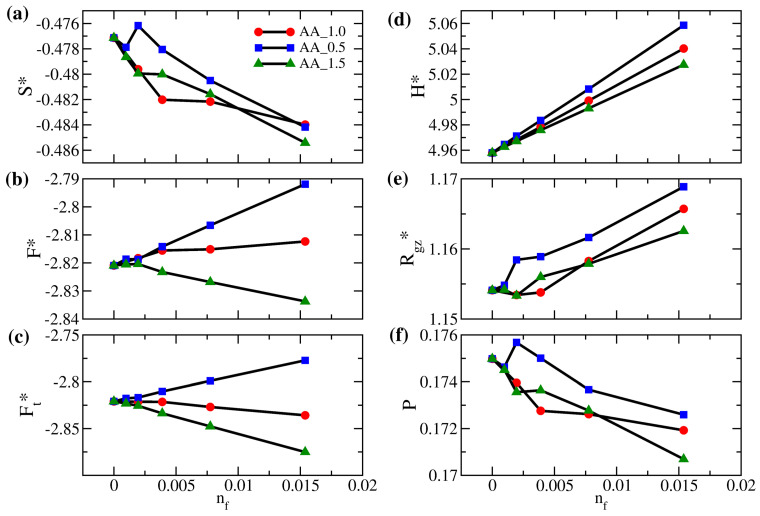
(**a**) Scaled entropy per bead ($S^{*}$), (**b**) scaled free energy per bead for polymer brushes ($F^{*}$), (**c**) scaled total free energy per bead ($F_{t}^{*}$), (**d**) scaled gap thickness ($H^{*}$), (**e**) degree of interpenetration of polymers (*P*), and (**f**) ratio of the *z*-component actual gyration radius to the reference gyration radius ($R_{gz}^{*}$) as a function of the number fraction of free particles ($n_{f}$) for brushes AA at $\varepsilon_{ff}=1$ (red circles), 0.5 (blue squares), and 1.5 (green triangles).

In Figure 9a,f, the variations in the interpenetration of the polymer brushes generally agree with the variations in entropy, consistent with the observations in Figure 7a,f. For the AA_0.5 system, as the free particles have a weaker attraction with the brushes and a weaker repulsion with the wall, the jump in the interpenetration and the sudden increase in $R_{gz}^{*}$ around $n_{f}=0.002$ are attributed to the increased packing of free particles near the grafting wall, which makes the brush stretch to the opposite wall. In contrast, for the AA_1.5 system over the same range of $n_{f}$, we observe a dip in both the interpenetration and $R_{gz}^{*}$ resulting from the stronger mixing between the free particle and the brush in the middle of the gap. We next compare the variations in the free energy per bead of the polymer brushes due to different affinities between the free particles and brushes in Figure 9b. For system AA_0.5, mixing between polymer brushes and free particles is less favorable, so the free energy per bead for the polymer brush generally increases. However, for system AA_1.5, the brush free energy increases at first due to the reduction in entropy. Eventually, it decreases as a result of the strong attraction between polymers and free particles. For a moderate amount of added particles, our simulation suggests that the enthalpic effect dominates for system AA_1.5. This competition between the entropic and enthalpic effects in different solute concentrations is unique to solvent-free brushes. Finally, as the overall system free energy is impacted by the miscibility of brushes and free solutes, it is not surprising that, when $n_{f}$ is higher, the total free energy per particle in Figure 9c increases for system AA_0.5 but decreases for AA_1.5.

Although the local volume fraction distributions of polymer brushes for systems AA_0.5, AA_1.0, and AA_1.5 in Figure 9a are nearly indistinguishable, the corresponding distributions of free particles in Figure 9b differ significantly. Compared to system AA_1.0, the two dominant peaks near the wall in the distribution of system AA_0.5 are more pronounced, which means that free particles are more likely to stay in the proximity of the two walls instead of staying in the polymer-rich middle region. Conversely, the two peaks disappear in system AA_1.5, where the monomer-solute attraction is the strongest. We observe a higher plateau in the central region, allowing the free particles to mix with the polymers uniformly. Making use of the total free energy of the system in the dilute limit ($n_{f}\to 0$), we present the variation of the solute chemical potential for the AA system in the inset of Figure 9b. For the range of $\varepsilon_{ff}$ considered, it is observed that the chemical potential is negative and decreases nearly linearly with $\varepsilon_{ff}$. This behavior suggests that the affinity between polymers and solutes sets the reference state of the solvent capacity for brushes. In contrast, the entropic driving force defines the deviation of the solvent capacity for the reference state.

**Figure 10:**
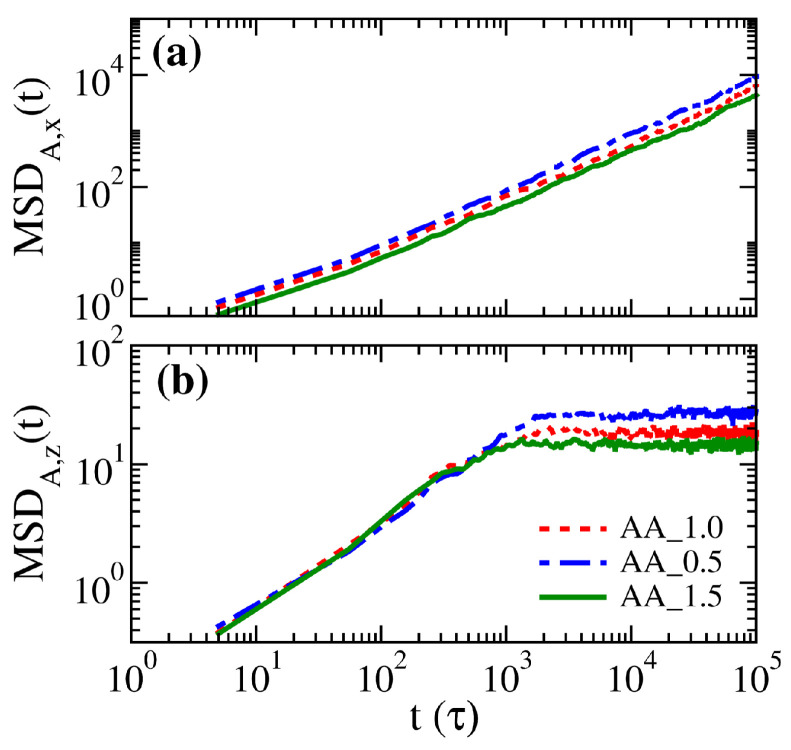
Mean square displacements of free particles in (**a**) the parallel direction and (**b**) the perpendicular direction for AA_0.5, AA_1.0, and AA_1.5 systems.

**Figure 11:**
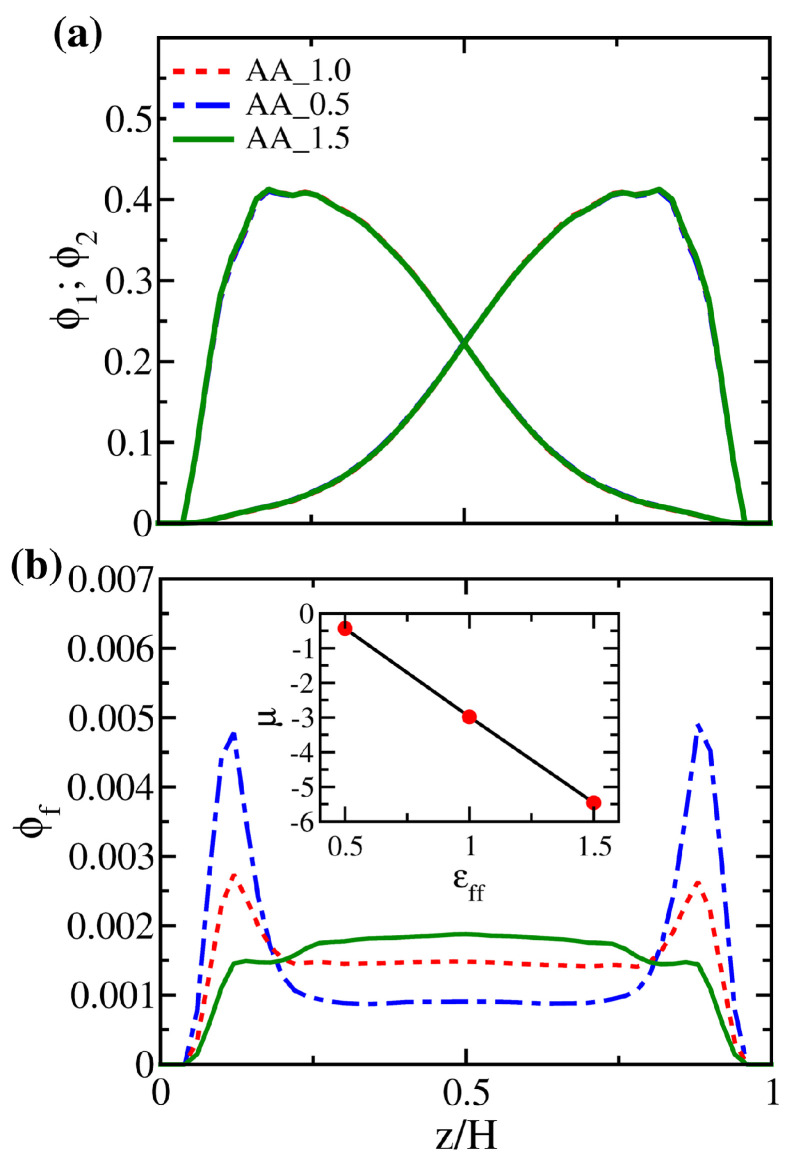
Local volume fraction distributions of (**a**) polymer beads and (**b**) free particles for systems AA_0.5, AA_1.0, and AA_1.5 at fixed $N_{f}=400$. The inset in (**b**) shows the variation in chemical potential (μ) for free particles in system AA as a function of $\varepsilon_{ff}$.

As the polymer distributions in systems AA_0.5, AA_1.0, and AA_1.5 do not show significant differences, it is expected that the corresponding segmental dynamics would again exhibit the same characteristics. Nevertheless, in Figure 10, the mean square displacements of free particles in the three brush systems exhibit variations. In the parallel direction, particles diffuse freely, and those with a weaker inter-particle attraction can diffuse faster. In the perpendicular direction, however, free particles roughly diffuse up to a mean square distance of 20, corresponding to the square of the distance between the peaks of the brush volume fractions in Figure 11a. This result suggests that the distribution of the brushes significantly limits the motion of free particles. Instead of diffusing unrestrictedly from one surface to the other across the gap, most free particles would be trapped in the middle region of the gap. Moreover, it is found that particles in the AA_0.5 system are more mobile as the weak attraction from the sea of polymer beads does not hinder their diffusion.

## 4. Conclusions

In this study, we investigated the role of enthalpic interactions in solvent-free interacting brushes using coarse-grained MD simulations. The LJ potential was used to model the monomer–monomer interaction, and the Berthelot mixing rule was applied for inter-species potential well depth. The enthalpic effects were elaborated through two model systems: one considering binary brushes of two different polymers and the other composed of single-component brushes mixed with free solute molecules. Given the interaction contrast between the two brushes based on the associated LJ potential well depths, we demonstrated that the miscibility between the two mismatched polymers is significantly improved when grafting on surfaces in solventless conditions. Meanwhile, as the brush with the weaker monomer–monomer attraction shows more extended chain configurations, the equilibrium inter-wall separation increases with the interaction contrast when the potential well depth of one of the brushes is fixed. Subsequently, we observe a generally lower degree of interpenetration between the brushes, and the corresponding segmental relaxation is facilitated as the attraction from the surrounding monomers is weaker.

The systems with different numbers of free particles inserted were used to examine the solution behavior of the brushes when external solute molecules were captured. To assess the capacity of the system for absorbing molecules, we calculated the chemical potential of free particles in the dilute limit. Two factors affect the solvent capacity: the chain configuration in the solvent-free state and the extent of LJ attraction between polymers and solutes. We found that the more entropically frustrated the polymers were, the stronger the driving force for the uptake of molecules. The solute distributions suggest that the absorption is driven by the solute–polymer mixing in the middle of the gap as well as the favorable packing in the free volume at the polymer–wall interface. Once a moderate amount of solutes are dispersed in the brushes, the enthalpic interaction dominates the overall sorption capacity between polymers and solutes. The mean square displacements of free molecules in parallel and perpendicular directions indicate that solutes diffuse freely tangential to the surface but are less mobile across the inter-wall space.

This study helps us understand the fundamentals of the enthalpic effect of mixing different kinds of NOHMs and the potential for capturing specific molecules using NOHMs. The entropic and enthalpic mechanisms of carbon capture outlined in the introduction are verified in this investigation. In the classical density functional theory [61], the entropic driving force is qualitatively predicted in terms of the intrinsic difficulty of the tethered polymers to fill the space. The current MD simulations move one step forward to identifying the source of the driving force by scrutinizing the distribution functions and the variations in the energy components in the presence of different fractions of solute molecules. Since the grafted polymers on the nanoparticle surface may alter the material properties, such as the configuration of grafted polymers and the corresponding chain relaxation dynamics, we anticipate that the simulation results presented may serve as a guideline for choosing the appropriate type of polymers for the material properties suitable for applications in gas sorption or carbon capture using NOHMs. Based on our developed simulation protocol, future investigations on the driving force for capturing different molecules may be undertaken.

## Figures and Tables

**Table 1:** Brush system classification. *N* is the number of monomers per chain, and $\varepsilon_{ii}$ is the LJ interaction strength between monomers in the brush.

Brush ID	*N*	$\varepsilon_{\mathrm{ii}}$
A	32	1.0
B	32	0.9
C	32	0.8
D	32	0.75
E	32	0.7
F	32	0.6
G	32	0.5
H	32	0.4
I	32	0.3
J	32	0.2
M	16	1.0

## Data Availability

The data supporting the findings of this study are available from the corresponding author upon reasonable request.

## Supplementary Materials

The following Supplementary Materials can be downloaded at: https://www.mdpi.com/article/10.3390/polym14235237/s1, Figure S1: (a) Scaled internal energy per bead ($U^{*}$), (b) scaled entropy per bead ($S^{*}$), (c) scaled free energy per bead ($F^{*}$), (d) degree of interpenetration of polymers (*P*), and (f) ratio of the *z*-component actual gyration radius to the reference gyration radius ($R_{gz}^{*}$) versus the dimensionless gap thickness ($H^{*}$) of AA, AB, and BB systems; Figure S2: Local volume fraction distributions of the polymer beads (ϕ) plotted along the scaled $z^{*}=z/R_{g}^{0}$ position for systems AA, AB, and BB. The distributions of the bottom brush are shown in (a) and those of the upper brush are in (b). The dashed curves in (a,b) correspond to the profiles of brush A and brush B in the binary system, respectively; Figure S3: Comparison of the normalized end-to-end fluctuation autocorrelation functions ($C_{A,\alpha }\left(t\right)$) for systems AA, AB, and BB in the parallel direction, (a), and the perpendicular direction, (b); Figure S4: Comparison of the mean square displacements of the end-to-end fluctuation (${MSF}_{A,\alpha }\left(t\right)$) for systems AA, AB, and BB in the parallel direction, (a), and the perpendicular direction, (b); Figure S5: Comparison of the probability distribution functions of the end-to-end fluctuation ($P(A_{\alpha })$) for systems AA, AB, and BB in the parallel direction, (a), and the perpendicular direction, (b); Figure S6: Comparison of the normalized end-to-end fluctuation autocorrelation functions ($C_{A,\alpha }\left(t\right)$) for systems of pure AA, AA with $N_{f}=400$, and AA with $N_{f}=1600$ in the parallel direction, (a), and the perpendicular direction, (b); Figure S7: Comparison of the mean square displacements of the end-to-end fluctuation (${MSF}_{A,\alpha }\left(t\right)$) for systems of pure AA, AA with $N_{f}=400$, and AA with $N_{f}=1600$ in the parallel direction, (a), and the perpendicular direction, (b); Figure S8: Comparison of the probability distribution functions of the end-to-end fluctuation ($P(A_{\alpha })$) for systems of pure AA, AA with $N_{f}=400$, and AA with $N_{f}=1600$ in the parallel direction, (a), and the perpendicular direction, (b); Table S1: Structural properties and energy components per bead of polymer brushes in system AA (i.e., AA_1.0) with different numbers of free particles; Table S2: Energy components of free particles per bead in system AA (i.e., AA_1.0); Table S3: Structural properties and energy components per bead of polymer brushes in system MM with different numbers of free particles; Table S4: Energy components of free particles per bead in system MM; Table S5: Structural properties and energy components per bead of polymer brushes in system AA_0.5 with different numbers of free particles; Table S6: Energy components of free particles per bead in system AA_0.5; Table S7: Structural properties and energy components per bead of polymer brushes in system AA_1.5 with different numbers of free particles; Table S8: Energy components of free particles per bead in system AA_1.5.
