# Supplementary material for: Enthalpic Interactions and Solution Behaviors of Solvent-Free Polymer Brushes
Source: Polymers (Basel). 2022 Dec 1;14(23):5237. doi: 10.3390/polym14235237 (PMC9740690; doi:10.3390/polym14235237)
Supplement: Supplementary file 1 [file polymers-14-05237-s001.zip › polymers-2042345-supplementary.pdf]

Supplementary Materials: Enthalpic Interactions and Solution Behaviors of Solvent-Free Polymer Brushes

Yi-Ju Chen and Hsiu-Yu Yu*

Department of Chemical Engineering, National Taiwan University,

No. 1, Sec. 4, Roosevelt Rd., Taipei 10617, Taiwan

E-mail: hsiuyuyu@ntu.edu.tw

S1 Results for the AA, AB, and BB systems

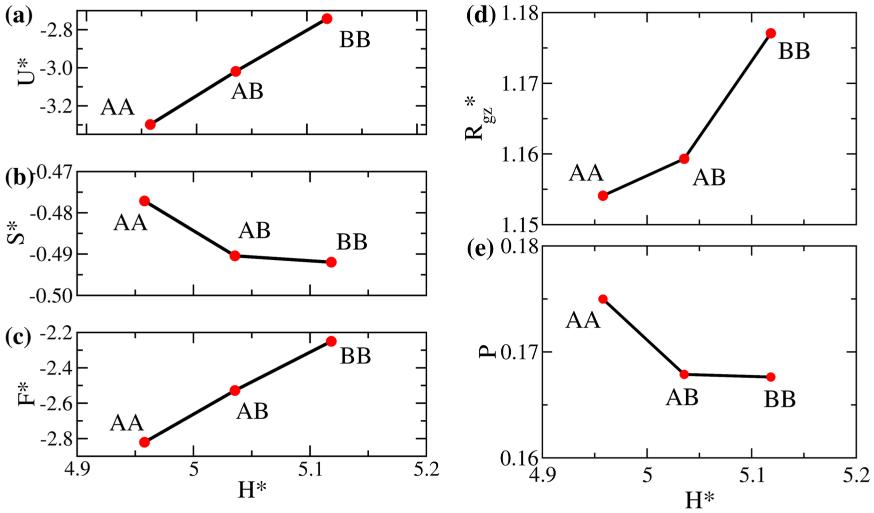

Figure S1: (a) Scaled internal energy per bead (U^*), (b) scaled entropy per bead (S^*), (c) scaled free energy per bead (F^*), (d) ratio of the z-component actual gyration radius to the reference gyration radius (R^*_{gz}), and (e) degree of the interpenetration of polymers (P) versus the dimensionless gap thickness (H^*) of AA, AB, and BB systems.

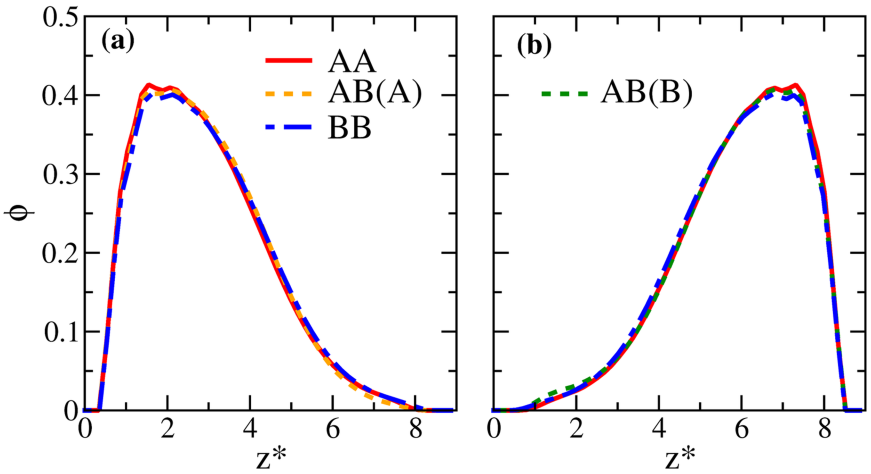

Figure S2: Local volume fraction distributions of the polymer beads (ϕ) plotted along the scaled $z^* = z/R_g^0$ position for systems AA, AB, and BB. The distributions of the bottom brush are shown in (a) and those of the upper brush are in (b). The dashed curves in (a) and (b) correspond to the profiles of brush A and brush B in the binary system, respectively.

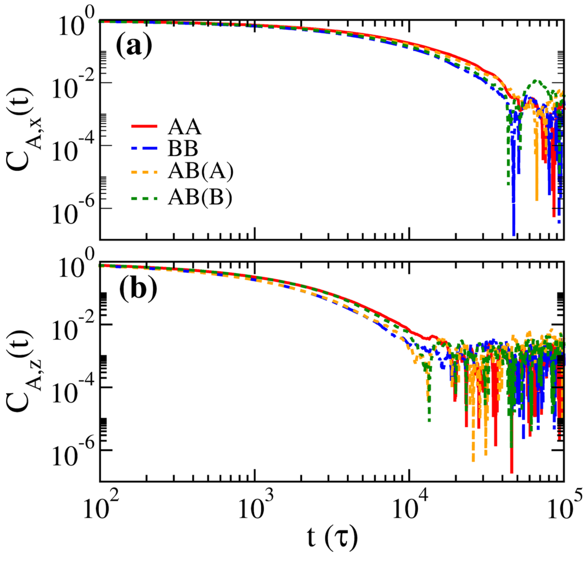

Figure S3: Comparison of the normalized end-to-end fluctuation autocorrelation functions ($C_{A,\alpha}(t)$) for systems AA, AB, and BB in the parallel direction, (a), and the perpendicular direction, (b).

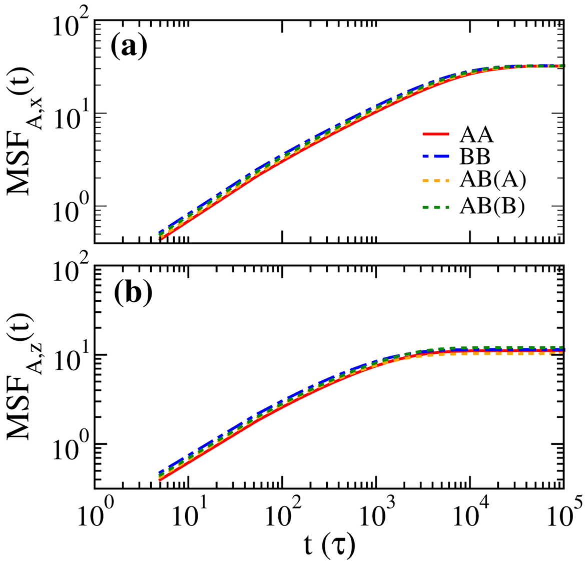

Figure S4: Comparison of the mean square displacements of the end-to-end fluctuation ($\mathrm{MSF}_{A,\alpha}(t)$) for systems AA, AB, and BB in the parallel direction, (a), and the perpendicular direction, (b).

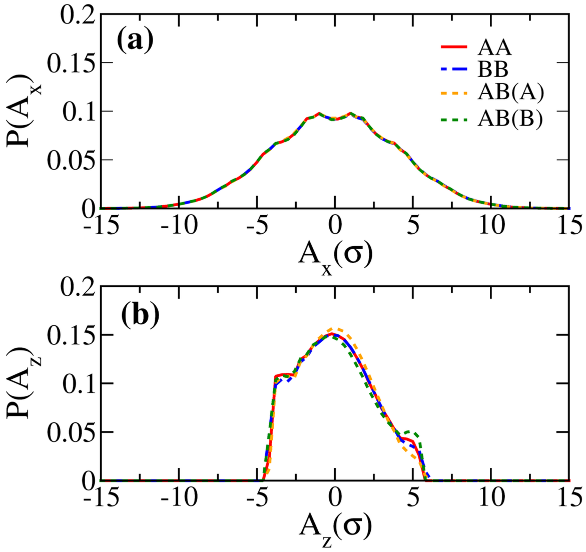

Figure S5: Comparison of the probability distribution functions of the end-to-end fluctuation ($P(A_\alpha)$) for systems AA, AB, and BB in the parallel direction, (a), and the perpendicular direction, (b).

S2 Dynamical results for the AA systems in the presence of free particles

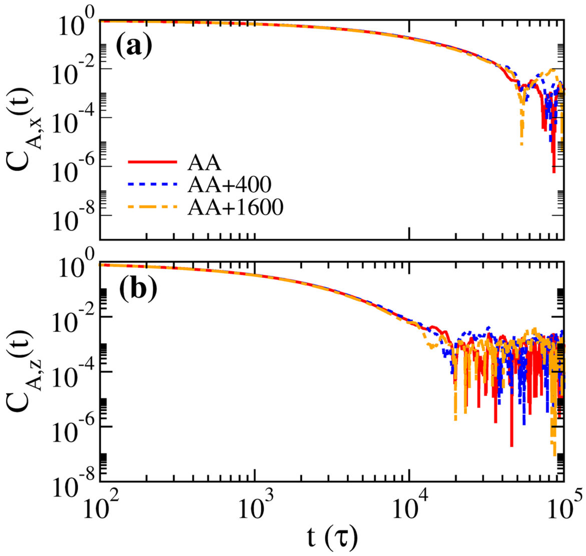

Figure S6: Comparison of the normalized end-to-end fluctuation autocorrelation functions ($C_{A,\alpha}(t)$) for systems of pure AA, AA with $N_f = 400$, and AA with $N_f = 1600$ in the parallel direction, (a), and the perpendicular direction, (b).

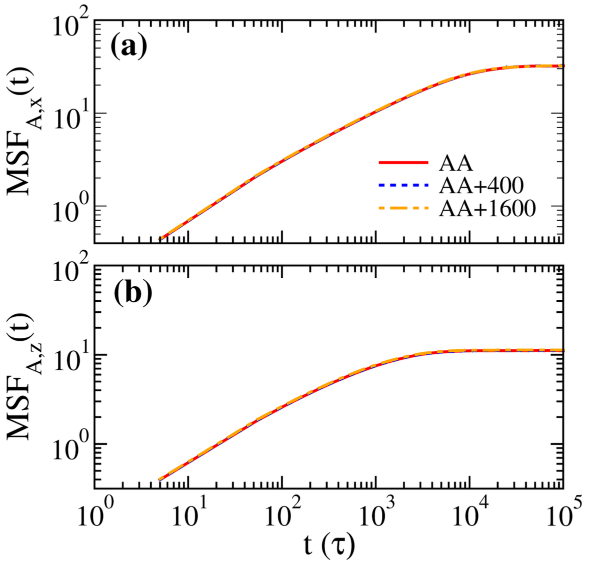

Figure S7: Comparison of the mean square displacements of the end-to-end fluctuation ($\mathrm{MSF}_{A,\alpha}(t)$) for systems of pure AA, AA with $N_f = 400$, and AA with $N_f = 1600$ in the parallel direction, (a), and the perpendicular direction, (b).

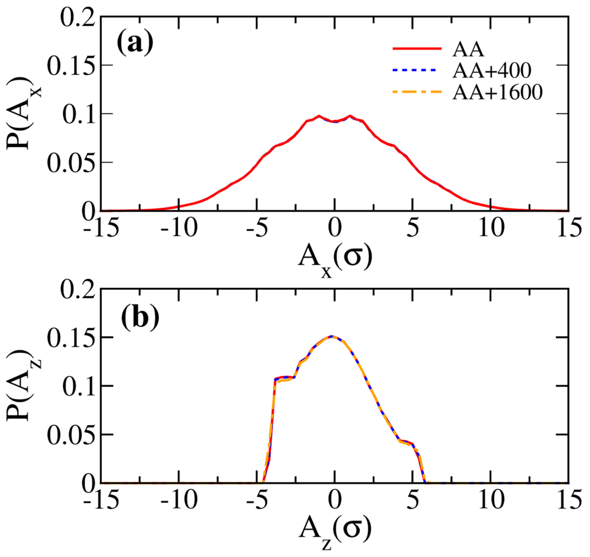

Figure S8: Comparison of the probability distribution functions of the end-to-end fluctuation ($P(A_\alpha)$) for systems of pure AA, AA with $N_f = 400$, and AA with $N_f = 1600$ in the parallel direction, (a), and the perpendicular direction, (b).

S3 Analyzed equilibrium data for solution behaviors

Table S1: Structural properties and energy components per bead of polymer brushes in system AA (i.e., AA_1.0) with different numbers of free particles.

N_f	$H(\sigma)$	H^*	S^*	U^*	F^*	P	R^*_{gz}
0	11.622	4.958	-0.477	-3.298	-2.821	0.175	1.154
200	11.646	4.968	-0.480	-3.298	-2.818	0.174	1.153
400	11.670	4.978	-0.482	-3.298	-2.816	0.173	1.154
800	11.719	4.999	-0.482	-3.297	-2.815	0.173	1.158
1600	11.815	5.040	-0.484	-3.296	-2.812	0.172	1.166

Table S2: Energy components of free particles per bead in system AA (i.e., AA_1.0).

N_f	n_f	S^*	U^*	F^*
200	0.00195	-0.205	-4.134	-3.929
400	0.00389	-0.205	-4.131	-3.926
800	0.00775	-0.205	-4.128	-3.923
1600	0.01538	-0.202	-4.135	-3.933

Table S3: Structural properties and energy components per bead of polymer brushes in system MM with different numbers of free particles.

N_f	$H(\sigma)$	H^*	S^*	U^*	F^*	P	R^*_{gz}
0	6.853	4.078	-0.511	-2.994	-2.4827	0.256	0.996
100	6.866	4.086	-0.512	-2.995	-2.4827	0.256	0.997
200	6.878	4.093	-0.512	-2.996	-2.4836	0.256	0.999
400	6.904	4.108	-0.515	-2.998	-2.4832	0.254	1.000
800	6.953	4.138	-0.518	-3.001	-2.4831	0.251	1.005

Table S4: Energy components of free particles per bead in system MM.

N_f	n_f	S^*	U^*	F^*
100	0.00195	-0.369	-3.676	-3.307
200	0.00389	-0.366	-3.687	-3.320
400	0.00775	-0.365	-3.681	-3.316
800	0.01538	-0.360	-3.693	-3.334

Table S5: Structural properties and energy components per bead of polymer brushes in system AA_0.5 with different numbers of free particles.

N_f	$H(\sigma)$	H^*	S^*	U^*	F^*	P	R^*_{gz}
0	11.622	4.958	-0.477	-3.298	-2.821	0.175	1.154
100	11.638	4.965	-0.478	-3.297	-2.819	0.175	1.155
200	11.653	4.971	-0.476	-3.295	-2.819	0.176	1.158
400	11.682	4.984	-0.478	-3.292	-2.814	0.175	1.159
800	11.740	5.008	-0.480	-3.287	-2.807	0.174	1.162
1600	11.858	5.059	-0.484	-3.276	-2.792	0.173	1.169

Table S6: Energy components of free particles per bead in system AA_0.5.

N_f	n_f	S^*	U^*	F^*
100	0.00098	-0.469	-2.307	-1.839
200	0.00195	-0.475	-2.299	-1.824
400	0.00389	-0.471	-2.299	-1.828
800	0.00775	-0.465	-2.295	-1.830
1600	0.01538	-0.455	-2.289	-1.834

Table S7: Structural properties and energy components per bead of polymer brushes in system AA_1.5 with different numbers of free particles.

N_f	$H(\sigma)$	H^*	S^*	U^*	F^*	P	R^*_{gz}
0	11.622	4.958	-0.477	-3.298	-2.8209	0.175	1.154
100	11.633	4.963	-0.479	-3.299	-2.8205	0.175	1.154
200	11.644	4.967	-0.480	-3.300	-2.8204	0.174	1.153
400	11.664	4.976	-0.480	-3.303	-2.8233	0.174	1.156
800	11.704	4.993	-0.482	-3.308	-2.8268	0.173	1.158
1600	11.785	5.027	-0.485	-3.319	-2.8337	0.171	1.163

Table S8: Energy components of free particles per bead in system AA_1.5.

N_f	n_f	S^*	U^*	F^*
100	0.00098	-0.183	-5.675	-5.492
200	0.00195	-0.184	-5.682	-5.498
400	0.00389	-0.183	-5.681	-5.498
800	0.00775	-0.183	-5.690	-5.508
1600	0.01538	-0.182	-5.706	-5.524
